# Research on a Segmentation Algorithm for the Tujia Brocade Images Based on Unsupervised Gaussian Mixture Clustering

**DOI:** 10.3389/fnbot.2021.739077

**Published:** 2021-09-03

**Authors:** Shuqi He

**Affiliations:** College of Computer Science, South-Central University for Nationalities, Wuhan, China

**Keywords:** Tujia brocade segmentation, GMM, DenseCRF, *K* auto-selection based on information fusion, optimization based on the vote

## Abstract

Tujia brocades are important carriers of Chinese Tujia national culture and art. It records the most detailed and real cultural history of Tujia nationality and is one of the National Intangible Cultural Heritage. Classic graphic elements are separated from Tujia brocade patterns to establish the Tujia brocade graphic element database, which is used for the protection and inheritance of traditional national culture. Tujia brocade dataset collected a total of more than 200 clear Tujia brocade patterns and was divided into seven categories, according to traditional meanings. The weave texture of a Tujia brocade is coarse, and the textural features of the background are obvious, so classical segmentation algorithms cannot achieve good segmentation effects. At the same time, deep learning technology cannot be used because there is no standard Tujia brocade dataset. Based on the above problems, this study proposes a method based on an unsupervised clustering algorithm for the segmentation of Tujia brocades. First, the cluster number *K* is calculated by fusing local binary patterns (LBP) and gray-level co-occurrence matrix (GLCM) characteristic values. Second, clustering and segmentation are conducted on each input Tujia brocade image by adopting a Gaussian mixture model (GMM) to obtain a preliminary segmentation image, wherein the image yielded after preliminary segmentation is rough. Then, a method based on voting optimization and dense conditional random field (DenseCRF) (CRF denotes conditional random filtering) is adopted to optimize the image after preliminary segmentation and obtain the image segmentation results. Finally, the desired graphic element contour is extracted through interactive cutting. The contributions of this study include: (1) a calculation method for the cluster number *K* wherein the experimental results show that the effect of the clustering number *K* chosen in this paper is ideal; (2) an optimization method for the noise points of Tujia brocade patterns based on voting, which can effectively eliminate isolated noise points from brocade patterns.

## Introduction

Intangible cultural heritage is an important symbol of the historical and cultural achievements of a country or a nation. It is not only of great significance to the study of the evolution of human civilization but also plays a unique role in showing the diversity of world culture, being the common cultural wealth of mankind. Tujia nationality is one of the 56 ethnic groups in China. Tujia brocade is an important carrier of the culture and art of Tujia nationality. Furthermore, it records the most detailed and real cultural history of Tujia nationality, making it one of the National Intangible Cultural Heritage (Wan and Nie, 2018).

The basic primitives of a Tujia brocade are extracted by digital image technology for classification and storage to form a Tujia brocade database. This provides a safe and convenient way to protect the Tujia brocade culture. Tujia brocades use cotton yarn as warps and silk thread or cotton thread, and useful wool, as wefts, which are much thicker than ordinary fabric fibers. Therefore, the weave texture of a Tujia brocade is coarse, and it is not easy to form smooth and round curves and shapes. Brocade patterns have pixelated visual textures and the features of abstract geometric patterns (Wan and Nie, 2018). These characteristics make Tujia brocade images have exceptionally large color characteristic differences from ordinary images, and the texture-level image contrast is not strong, which brings difficulty to image segmentation.

Image segmentation is one of the research hotspots in the field of computer vision. The traditional image segmentation algorithms mainly use the low-level semantics of images including color, texture, and shape for segmentation, such as threshold method, region grow algorithm, and edge detection algorithm, among others (Heath et al., 1997; Fan et al., 2001; Otsu, 2007). Superpixel segmentation methods emerged after researchers introduced graph theory to image segmentation such as Graph Cuts and Simple Linear Iterative Clustering (SLIC) (Felzenszwalb and Huttenlocher, 2004; Achanta et al., 2012). It is difficult to achieve semantic segmentation *via* traditional clustering segmentation based on the shallow features of images.

The model based on deep learning can automatically extract the image features representation and has achieved excellent results in many challenging computer vision tasks, including object detection, location, recognition, and segmentation. Classic image segmentation models such as Fully Convolutional Networks (FCN) (Long et al., 2015), Mask Regional-Based Convolutional Neural Networks (Mask R-CNN) (He et al., 2017), DeepLab, and so on. The semantic segmentation DeepLab (Chen et al., 2018a,b) employs a series algorithm by integrating various classical deep learning methods and using Atrous Convolution, Atrous Spatial Pyramid Pooling (ASPP), along with the other structures. Meanwhile, a dense conditional random field (DenseCRF) structure was connected to the back end of the neural network to provide a refined segmentation for the boundary after initial segmentation. Nonetheless, most classic image segmentation models rely on high-quality massive datasets. It is difficult to conduct image segmentation by the classic deep learning segmentation model because the dataset in this study only contains more than 200 images without a pixel-level segmentation tag.

More recently, unsupervised deep learning becomes a research hotspot. A dual-branch combination network (DCN) (Yang et al., 2017) was proposed as a method combining an autoencoder and *K*-means. The model encoder maps the input data from high-dimensional features to low-dimensional subspaces, obtains the potential features of the data, performs *K*-means clustering on them, and obtains the *K*-means loss. The decoder reconstructs the latent features into the original data to obtain the reconstruction loss. The network combines the reconstruction loss and *K*-means loss through backpropagation to optimize the learning process. The study of Kanezaki (2018) used standard unsupervised over-segmentation techniques to supervise convolutional neural networks. This method uses standard algorithms to extract pre-segmented regions from the original image. The segmentation model extracts image features through convolutional neural networks to obtain a rough segmentation of the image and then adjusts the rough segmentation results according to multiple constraints, such as feature similarity and spatial continuity so that all pixels in the same pre-segmented area have the same label. The loss incurred between the two segmentation images before and after the adjustment is used as the backpropagation loss of the supervision signal to update the network weight.

The recognition and segmentation of brocade texture are also one of the applications of image segmentation. Brocade texture feature extraction technology originated in the mid-1980's. Over the past decade, researchers began to focus on textile-aided design, fabric pattern segmentation, and contour extraction technology. The study of Kuo et al. (2005, 2007) and Kuo and Shih (2011) advocated extracting the color features of printed fabrics through feature extraction algorithms, such as self-organizing map network (SOM), and then obtained the pattern by using the Fuzzy-c means (FCM) algorithm to achieve the automatic classification of the colors. The study of Lachkar et al. (2006) adopted a clustering method based on a GMM. The method combined a GMM and a content validity index (CVI) to form an adaptive, efficient segmentation algorithm. In the research conducted by Jiang et al. (2014), they studied the automatic recognition technology of jacquard warp knitted fabric pattern images. The fabric image uses a two-dimensional wavelet decomposition algorithm to extract features, given the clustering center, and then uses the *K*-means clustering multi-channel algorithm for segmentation.

Based on the research of textile image segmentation algorithm, we found that there are two difficulties in the segmentation of Tujia brocade by the commonly used image segmentation algorithm.

- The material of Tujia brocade is rougher than the common fabric fiber and the background texture of the brocade pattern is very prominent. This forms a similar feeling to “mosaic,” which is represented as a noise signal on the fabric image. Such kind of noise information can cover up part of the detail information, and increase the image entropy, making the boundary between the Tujia brocade primitive and the image background becomes blurred. This will increase the difficulty of edge detail segmentation and reduce the accuracy of pattern texture segmentation.
- Deep learning-based image segmentation algorithms typically use large datasets for training to prevent overfitting during data processing. Tujia brocade image segmentation research is relatively rare; there is a lack of training data specifically designed for brocade image segmentation. If the image matting or image segmentation tools are used to build a data set, it needs a lot of manual labor to extract material from massive data through tedious operations.

In response to the above problems, this study proposes a clustering segmentation process for Tujia brocades. First, the input Tujia brocade is divided into basic clusters. Then, a voting-based optimization method is used to eliminate the noise points of the image based on the characteristics of the Tujia brocade. Afterward, DenseCRF is employed to optimize the image and obtain effective segmentation results. Finally, the desired primitive outline is extracted through interactive cutting. The contributions of this study are as follows:

- A calculation method for cluster number *K*. In unsupervised clustering, the *K*-value has an extraordinarily strong impact on the clustering results. The algorithm uses local binary patterns (LBP) to calculate the base for the image texture features and uses the feature value of the GLCM as the weight. The two values are fused to calculate the *K*-value for clustering. Experiments show that the clustering effect of the *K*-value selection algorithm is ideal.
- An optimization method for Tujia brocade noise points. Due to the extensive weave textures of Tujia brocades and the obvious textural characteristics of the background, noise points easily occur after clustering. DenseCRF can be used to optimize the image contour, but it is not effective in eliminating the noise points of a Tujia brocade. Therefore, we propose a voting-based optimization method. The classification labels obtained after the preliminary clustering process are voted on according to the classification results of their neighboring pixels to redistribute the labels of the center pixels. This method for the elimination of isolated noise points is remarkably effective and is then combined with DenseCRF to optimize the preliminary clustering-based segmentation map to obtain the final Tujia brocade segmentation map.

## Method

**Figure 1:**
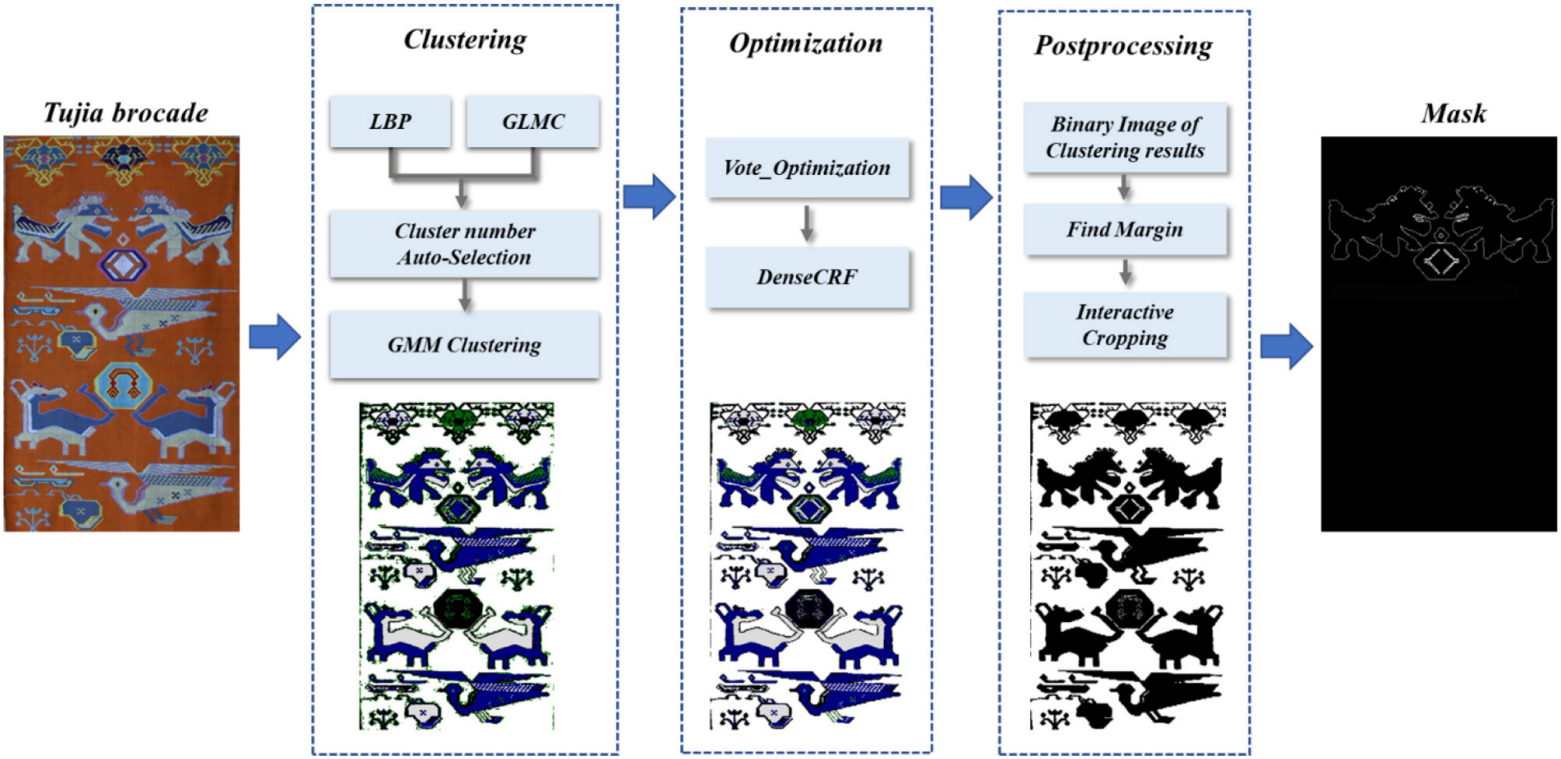
Tujia brocade image segmentation process.

For a small unlabelled dataset, we used an unsupervised clustering method to segment the input Tujia brocade. First, the LBP and GLCM feature values were fused to calculate the *K*-value of the cluster. Afterward, a GMM is used to cluster and obtain a preliminary segmentation map. This approach does not extract image features that are different from those obtained *via* traditional image segmentation. The image yielded after the initial segmentation process is relatively rough, and we propose a method based on the combination of voting optimization and DenseCRF to optimize this to obtain the final image segmentation result. The specific flow chart is shown in Figure 1.

### Cluster Number *K* Auto-Selection

**Figure 2:**
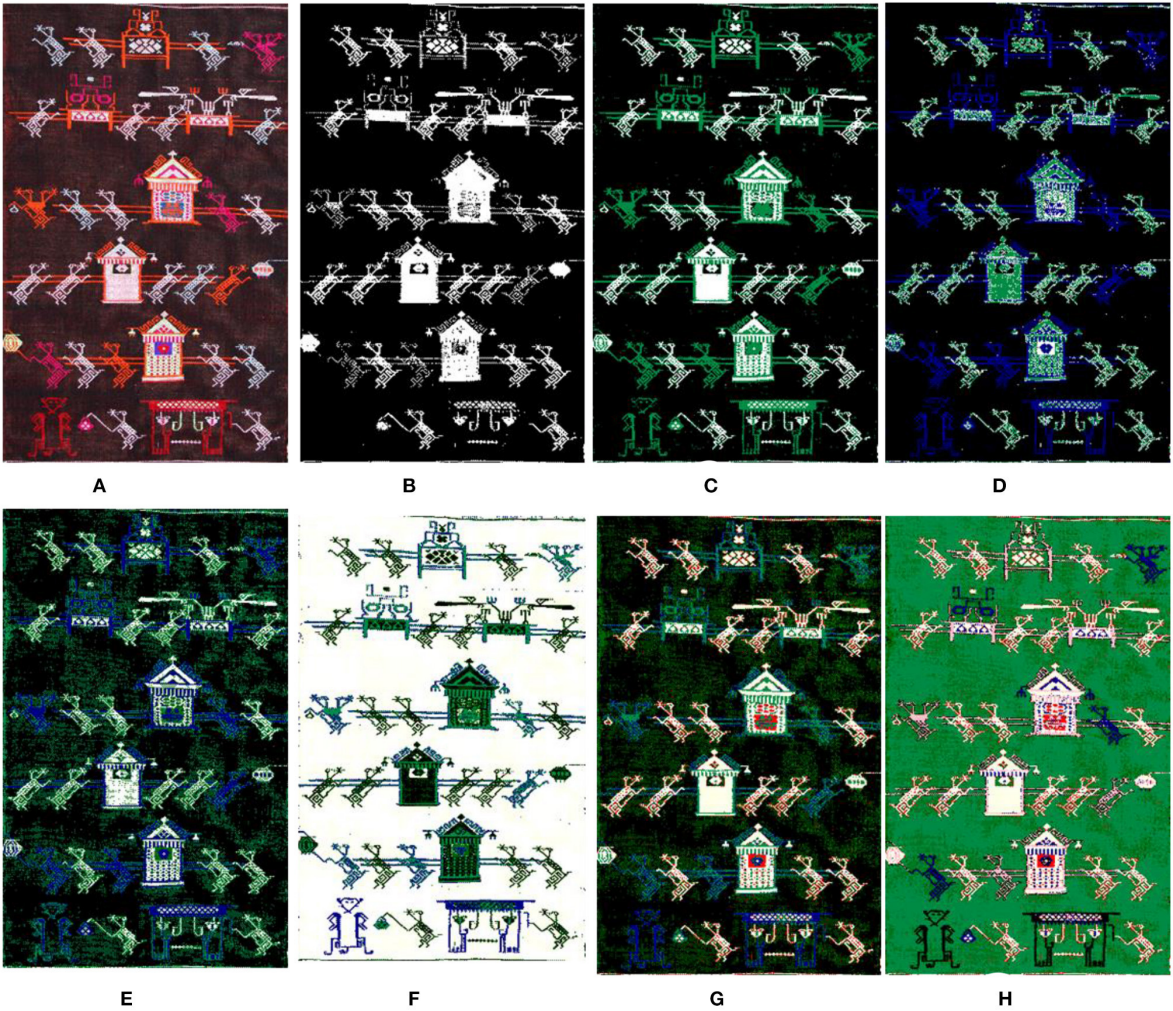
The segmentation results by the GMM uses different *K*-values. **(A)** Original. **(B)** *K* = 2. **(C)** *K* = 3. **(D)** *K* = 4. **(E)** *K* = 5. **(F)** *K* = 6. **(G)** *K* = 7. **(H)** *K* = 8.

In an image, regions belonging to the same object mostly have similar textures and colors. During the image clustering segmentation, similar pixels were classified into a category. This category is regarded as a segmentation object which is classified according to the similarity between image pixels. The *K*-value selection is particularly important to obtain a good image segmentation effect. Due to the influence of brocade weaving technology, the image background of Tujia brocade has a strong sense of grain. If the *K*-value is too large when clustering, the image background will be clustered, forming the mosaic effect and affecting the segmentation effect. However, if the *K*-value is too small, the fine lines in the image will be ignored. Figure 2 shows the segmentation effect of different *K*-values in the GMM algorithm.

Under observation, we found that the visual effect of the clustering was better when *K* = 2, 3, or 6, but we were not sure exactly what the clustering *K*-value should be until the clustering results come out. The model was selected mostly through criterion functions such as Bayesian information criterion (BIC) (Chakrabarti and Ghosh, 2011), Akaike information criterion (AIC) (Burnham and Anderson, 2002), among others. However, such application was very difficult in the actual model selection because the computational effort was too large, and it was found *via* specific experiments that the model selected by the criteria function was not the optimal estimation model for the image segmentation. All models obtained by training were only regarded as an approximate model of the real model. The objective of this study is to obtain a reasonable clustering *K*-value quickly and effectively. Traditional Tujia brocade consists of many similar graphic elements with strong regularity and has obvious texture features. For this reason, the number of texture features can be used to select the *K*-value of the clustering model. We introduce the statistical eigenvalues of the image GLCM and LBP to calculate the *K*-value.

#### Local Binary Patterns

Local binary patterns is an operator to describe the local texture features of the image and has gray and rotation invariance. LBP operator proposed by the study of Ojala et al. (2002) can divide the whole image into different subregions to perform local texture feature histogram statistics in each small region, that is, to count the feature number belonging to a certain pattern in the region. Finally, the histogram of all regions was connected as the image feature vector. The original LBP operator took the center pixel of the 3 × 3 window as the threshold value to compare the gray values of the adjacent eight pixels with the threshold value in turn clockwise. If the gray value is greater than or equal to the threshold value, the value of this pixel point is marked as 1, otherwise 0. After the comparison between the adjacent eight pixels, an 8-bit binary number was generated as the LBP value of the center pixel of the window to reflect the texture information of the region. The specific calculation process is shown in Formula (1).

(1) $$\begin{array}{l} LBP\left(x_{c},y_{c}\right)=\overset{p-1}{\underset{p=0}{\sum }}2^{p}s\left(i_{p}-i_{c}\right). \end{array}$$

where (*x*_*c*_, *y*_*c*_) is the coordinate of the central pixel; *p* is the *p*^th^ pixel of the adjacent region; *i*_*p*_ is the gray value of the pixel of the adjacent region; *i*_*c*_ is the gray value of the central pixel; *s(x)* is a sign function as shown in Formula (2).

(2) $$S\left(x\right)=\{\begin{array}{c} 1,\;if\;x\geq 0\\ \;\\ 0,\;else \end{array}$$

The original LBP operator only covers a small area of 3 × 3 in practical application, which cannot adapt to the texture features of different sizes. For this purpose, Extended LBP (Ojala et al., 2002) was proposed which extended the coverage area of the LBP operator to a circular neighborhood with a radius of *R*. The LBP operator can sample P points in the circular region. The method adopted Uniform Pattern LBP. P sampling points generated 2^P^ patterns in Extended LBP. The introduction of “equivalent mode” (Ojala et al., 2002) reduced the number of modes from the original 2^P^ to P(P – 1) + 2. We adopted the LBP algorithm which can calculate the occurrence frequency of image texture feature pattern, to calculate the cardinality of clustering *K*-value.

#### Gray Level Co-occurrence Matrix (GLCM)

Tujia brocade images are generally permuted by many repeated arrays of basic primitives. The basic texture feature cardinality calculated by the LBP operator may not fully represent the number of categories of segmented objects. Therefore, we introduced the statistical feature values of the image GLCM (Sulochana and Vidhya, 2013) which was commonly used to describe texture by studying the spatial correlation characteristics of gray level. The texture is formed by the repeated appearance of gray distribution in spatial positions, so there is a certain gray relationship between two pixels separated by a certain distance in the image space, that is, the spatial correlation characteristics of gray level in the image. For GLCM, the joint probability density of the two pixels was used to reflect the gray direction, interval, and change amplitude of the image. However, GLCM cannot directly provide the features of the texture. Some scalars can be used to represent GLCM features. The entropy value of the co-occurrence matrix contains the randomness measure of the image information amount, indicating the complexity of the image gray level distribution. The greater the entropy value is, the more complex the image is, as shown in the calculation Formula (3). The *M*-value reflects the degree of regularity of the texture. The smaller *M-*value means that the texture features are more chaotic and difficult to describe, as shown in the calculation Formula (4). The greater the contrast of the image, the clearer the visual effect of the image, as shown in the calculation Formula (5). We assumed that images with more complex patterns and chaotic texture features tended to be described by more models.

(3) $$\begin{array}{l} Entropy=-\overset{L-1}{\underset{i=0}{\sum }}\overset{L-1}{\underset{j=0}{\sum }}P\left(i,j,d,\theta \right)\times \ln\;P\left(i,j,d,\theta \right) \end{array}$$

(4) $$\begin{array}{l} Mean=\overset{L-1}{\underset{i=0}{\sum }}\overset{L-1}{\underset{j=0}{\sum }}P\left(i,j,d,\theta \right)\times i \end{array}$$

(5) $$\begin{array}{l} Contrast=\overset{L-1}{\underset{i=0}{\sum }}\overset{L-1}{\underset{j=0}{\sum }}p\left(i,j\right)\times {\left(i-j\right)}^{2} \end{array}$$

#### Calculating K-Values

The occurrence frequency of LBP texture features in the image was counted by the algorithm where a threshold value was set up and the number of LBP features whose frequency exceeds the threshold value was used as the cardinality of clustering *K*-value. Entropy, *M*, and contrast parameters of GLCM were used to calculate the weight of the clustering *K*-value. The calculation formula of *K*-value was shown as Formula (6). The weight calculation formula of clustering *K*-value was shown as Formula (7).

(6) $$\begin{array}{l} K=COUNT\left(P\left(LBP_{image\text{\_}i}\right)>threshold\right)\times W_{image\text{\_}i} \end{array}$$

(7) $$\begin{array}{l} W_{image\text{\_}i}=Entropy\times \alpha_{1}+Mean\times \alpha_{2}+Contrast\times \alpha_{3} \end{array}$$

In Formula (6), *P(LBP*_*image*_*i*_*)* represents the frequency of a texture feature; *W*_*image*_*i*_ represents the image texture complexity measure of *image*_*i*, which is obtained by Formula (7).

### Gaussian Mixture Model

The GMM (Bishop, 2006) is a probabilistic model. In image segmentation, image features, such as gray information, color information, or texture information, are used as the observation vectors of the image. It is assumed that the overall image pixels obey a Gaussian mixture distribution. The segmented areas can be regarded as single Gaussian models with the same form, and each model is independent of all other models. The entire image is a GMM formed by fusing multiple single Gaussian models with a certain weight.

Assuming that the GMM is composed of *K*-Gaussian models (the data contain *K*-classes), the probability density function of the GMM is shown in Formula (8) (Bishop, 2006).

(8) $$\begin{array}{l} p\left(x\right)=\overset{K}{\underset{k=1}{\sum }}w_{k}g\left(x|\mu_{k},\sum_{k}\right)\; \end{array}$$

where *K* is the number of components in the GMM, *w*_*k*_ is the mixture weight, which represents the proportion of the *K* single Gaussian models in the mixture model, 0 ≤ *w*_*k*_ ≤ *1*, $\overset{K}{\underset{k=1}{\sum }}w_{k}=1$, *g*(*x*|μ_*k*_, ∑_*k*_) is the distribution of the Gaussian component *k*, and its function expression is shown in Formula (9) (Bishop, 2006).

(9) $$\begin{array}{l} g\left(X\right)=\frac{1}{\sqrt{{\left(2\pi \right)}^{N}|\sum |}}e^{-\frac{1}{2}{\left(X-\mu \right)}^{T}\sum^{-1}\left(X-\mu \right)}\; \end{array}$$

where X is a random variable (which can be understood as the observation vector of the image), N is an arbitrary integer determined by the dimensionality of X, μ is the mean vector, $\mu =E\left\{X\right\}={\left[\mu_{1},\mu_{2},\cdots \;,\mu_{N}\right]}^{T}$, ∑ is the covariance matrix, N × N represents the number of dimensions, ∑^−1^ is the inverse matrix of ∑, and |∑| is the determinant of ∑, ∑ = *E*{(*X* − μ)(*X* − μ)^*T*^}. The iterative EM algorithm is used to solve the likelihood function criterion of the GMM and estimate the Gaussian distribution parameters to obtain the probability that each pixel belongs to each category. Finally, the category with the highest probability is regarded as the category to which the pixel belongs; this process is repeated until all image pixels have been classified, thus realizing the segmentation of the entire image. The likelihood function criterion is shown in Formula (10) (Bishop, 2006).

(10) $$\begin{array}{l} L\left(\theta \right)=ln\left[\underset{i=1}{\overset{n}{\prod }}p\left(x\right)\right]=\overset{n}{\underset{i=1}{\sum }}ln\overset{K}{\underset{k=1}{\sum }}w_{j}g\left(x|\mu_{k},\sum_{k}\right)\; \end{array}$$

### Optimization of the Clustering Results

#### Optimization Method Based on the Voting Method

**Figure 3:**
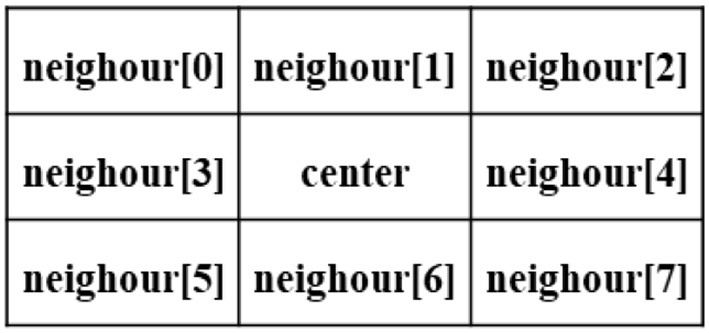
Optimization based on a voting window.

**Figure 4:**
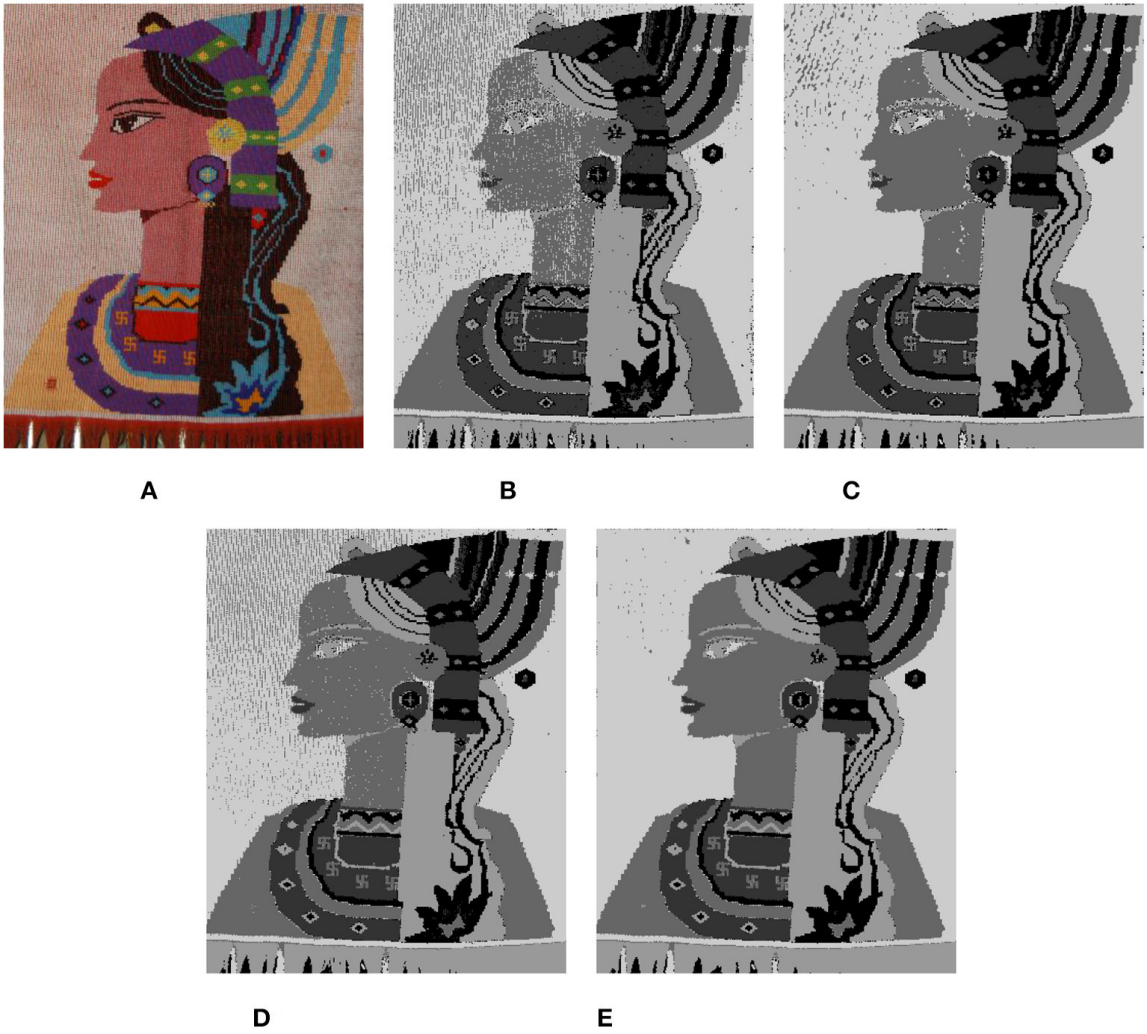
Optimization of clustering results. **(A)** Image: Original. **(B)** GMM, *K* = 5. **(C)** Vote_Optimization: GMM + Vote_Optimization. **(D)** GMM + DenseCRF. **(E)** GMM + DenseCRF + Vote_Optimization.

Traditional Tujia brocades use cotton yarn, silk thread, or cotton thread as the main weaving materials, and the formed image background has a strong weave texture, as shown in Figure 4A. After clustering, some noise points are formed that affect the segmentation results, as shown in Figure 4B. GMM clustering yields the classification probabilities of image pixels. Clustering does not consider the relationships between image pixels, and misclassification occurs when the image quality is not high. Generally, adjacent pixels in an image may belong to the same object. We draw on the idea of voting and define a 3 × 3 window, as shown in Figure 3. The center pixel is reassigned to a category according to the classification probabilities of the adjacent eight pixels. The algorithm sets a threshold, and when the probability of a category among the eight pixels adjacent to the center pixel exceeds the threshold, the category of the center pixel is modified to this category.

Each *neighbor[i]* has two attributes (Prob, label), where the label represents the assigned category *k* for the pixel, *Prob*=*[P(2), P(3), ……, P(k)], k* ∈ *[2, K]*, and *P(k)* represents the probability that this pixel belongs to category *k*. The category assignment of the center pixel is calculated *via* Formula (11).

(11) $$\begin{array}{l} Prob\left[k\right]=\underset{k\in \left(1,K\right)}{\max}\left(Average\overset{8}{\underset{i=1}{\sum }}Prob\left(neighour\left[i\right]\right)\right) \end{array}$$

If *Prob[k]* > = *threshold*, then *label[center]* = *k*.

Experiments show that this optimization process has a good effect on eliminating obvious independent noise points. As shown in Figure 4C, after the algorithm iteratively optimizes the image once, the background lines and noise points evidently disappear.

#### Dense Conditional Random Field

Optimization based on the voting method considers only the associations between neighboring pixels without considering the overall image and cannot optimize the image globally. As shown in Figure 4C, the details of the clustering result are relatively rough. For further optimization, we introduce DenseCRF. If the distance between and colors of the image pixels are very close, they belong to the same category in theory. DenseCRF (Philipp and Koltun, 2012) readjusts the existing clustering results from these two aspects based on the colors and the spatial location information of the pixels provided by the entire image and assigns the attributes of the pixels. In the fully-connected random field, the energy function of label *x* is expressed as Formula (12) (Philipp and Koltun, 2012).

(12) $$\begin{array}{l} E\left(x\right)=\underset{i}{\sum }\theta_{i}\left(x_{i}\right)+\underset{ij}{\sum }\theta_{ij}\left(x_{i},x_{j}\right) \end{array}$$

In the formula, the unary potential θ_*i*_*(x*_*i*_*)* comes from the front-end output (such as predicted by a classifier), and it represents the energy of dividing pixel *i* into label *x*_*i*_, which includes the shape, texture, position, and color of the image. The pairwise potentials θ_*ij*_*(x*_*i*_*, x*_*j*_*)* is the energy in which the pixel *i* and *j* are simultaneously assigned label *x*_*i*_ and *x*_*j*_. It describes the relationship between the pixels and encourages similar pixels to be assigned the same label. Pixels with large differences are assigned different labels so that the model can segment the image at the boundary as much as possible.

As shown in Figure 4D, the details of the clustering results are more delicate and smoother after DenseCRF optimizes the clustering results, but there are still background textures and noise points. We combine the two optimization methods, and the final optimization result is shown in Figure 4E.

### Mask Extraction

**Figure 5:**
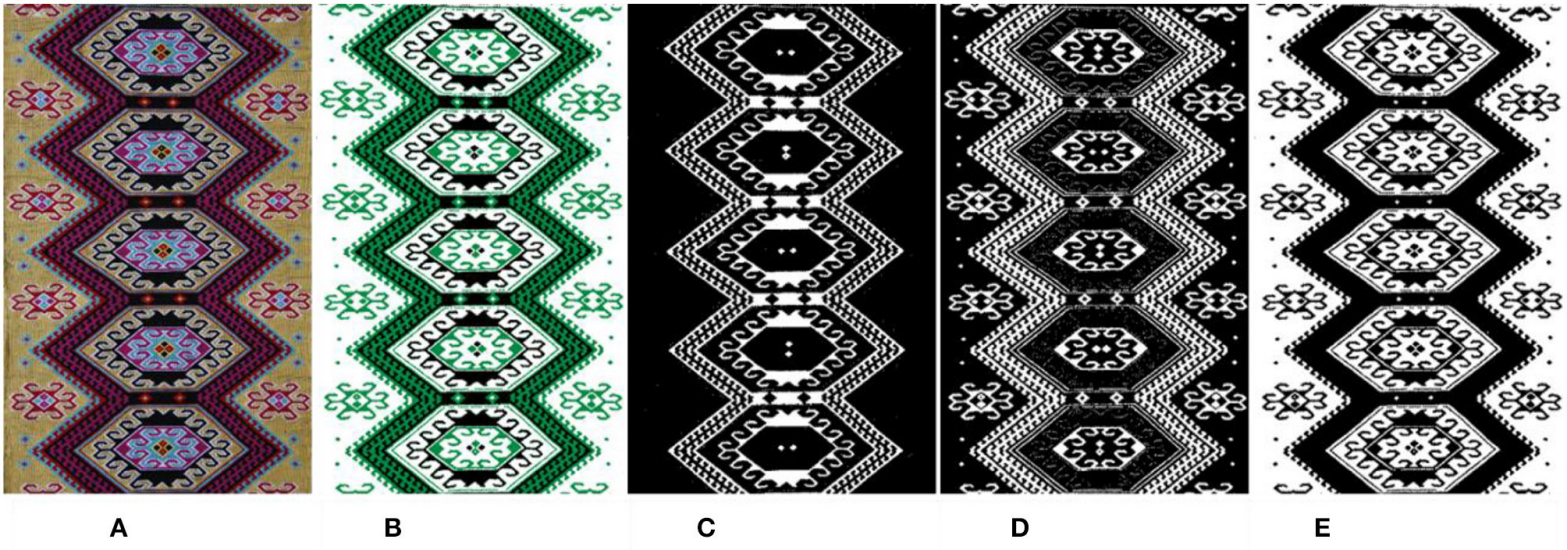
Binarized images of the clustering categories. **(A)** Image: Original. **(B)** Cluster (*k* = 3). **(C)** Binary Image (Label = 0). **(D)** Binary Image (Label = 1). **(E)** Binary Image (Label = 2).

**Figure 6:**
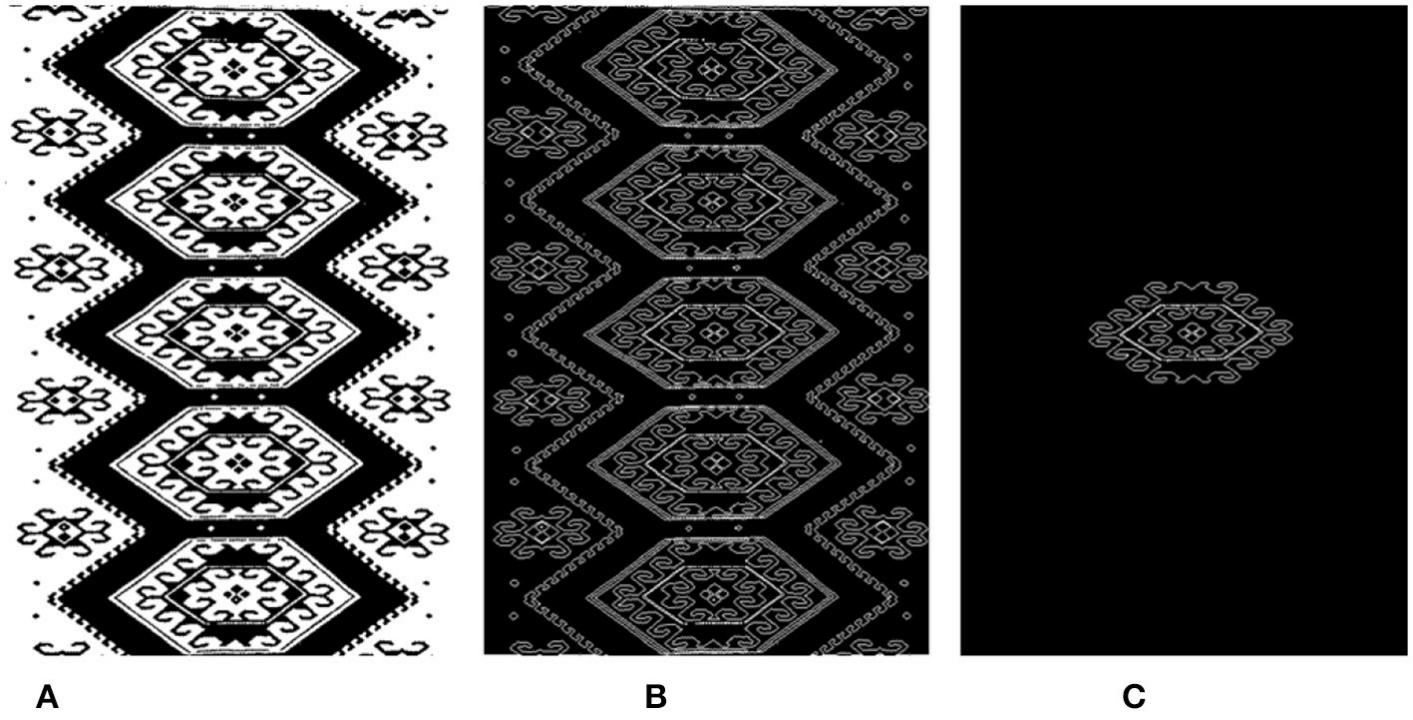
Image mask obtained by interactive cutting. **(A)** Binary Image (Label = 2). **(B)** Contour. **(C)** Mask.

A Tujia brocade is a geometric lattice pattern. Because of the interweaving of warps and wefts, its patterns are mostly composed of parallel lines, vertical lines, and diagonal lines. The clustering results in Figure 5B are shown in Figures 5C–E, which correspond to the binarized images (black background) of the clustering categories (such as label = 0 and label = 1). Each binary image (as Figure 6A) can be regarded as a part of the texture object that needs to be extracted, and its contour (as Figure 6B) is detected for interactive segmentation to obtain the object mask, as shown in Figure 6C.

## Experiments

The experiment is implemented by Python Software Foundation and the experimental environment is Microsoft Windows 10. The testing machine contains an Intel Core i7-8750H 2.20 GHz, an Nvidia GeForce GTX 1060 with Max-Q Design, and 24 GB of memory.

### Dataset

**Figure 7:**
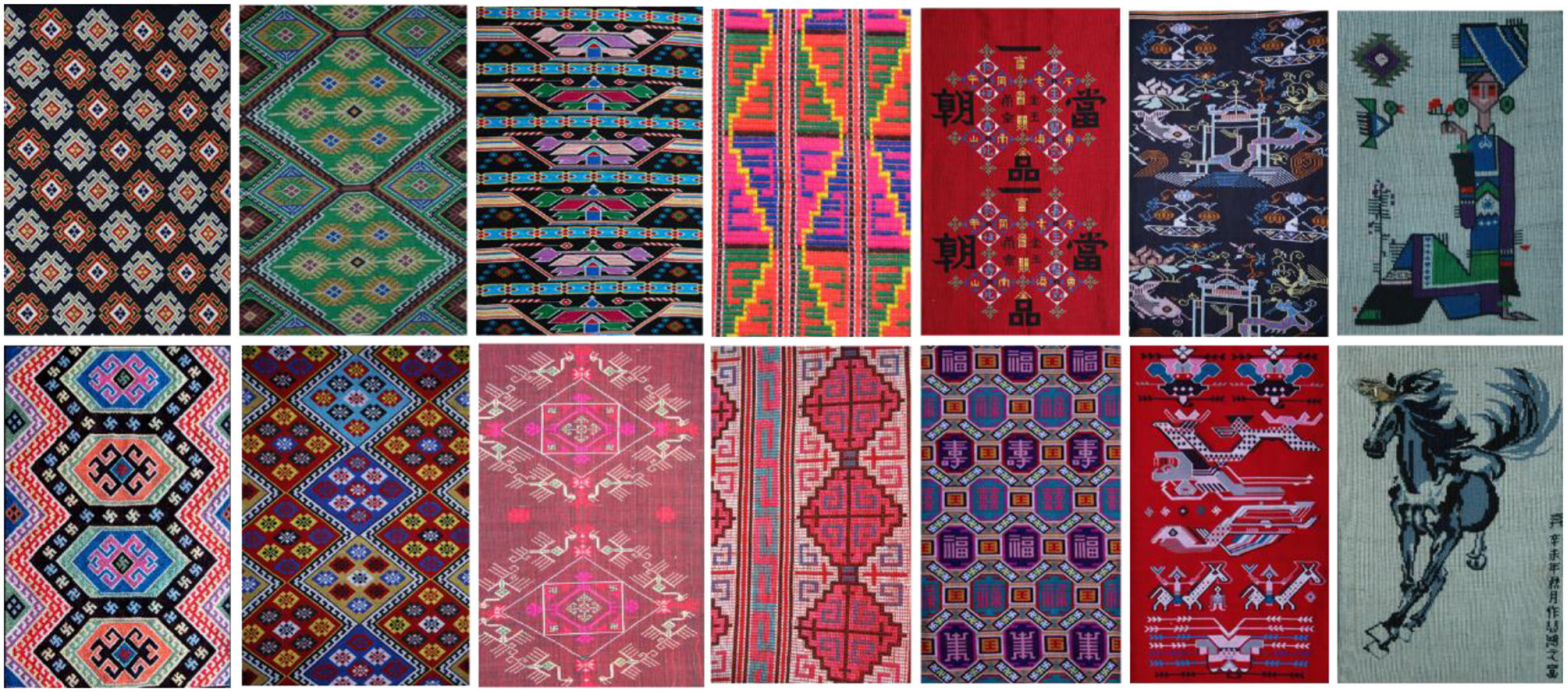
Tujia brocade dataset.

Since there are few studies on Tujia brocade image segmentation based on machine learning, there is no ready-made Tujia brocade dataset for use in experiments. We retrieve public Tujia brocade image data from the Internet, manually photograph the Tujia brocade, and collect a total of more than 200 clear Tujia brocade patterns. According to the traditional meanings of the Tujia brocades, the patterns are roughly divided into six categories: animals patterns, flowers and plants patterns, living utensils patterns, natural object patterns, geometric patterns, text patterns. The woven material of a Tujia brocade is rougher than ordinary fabric fibers, so the background textures of the brocade patterns are very prominent, the pictures are not clear, and the brocades are bright in color, as shown in Figure 7.

### Selection of the Clustering Value *K* and Evaluation of the Clustering Results

#### Evaluation by the Elbow Method

In unsupervised clustering, the clustering effect on the image details becomes clearer as the *K*-value increases, which is due to the particularity of the Tujia brocade dataset. When *K* reaches a certain critical point, the definition of the image details increases. However, the background texture is also clustered, forming noise points that affect the clustering results.

**Figure 8:**
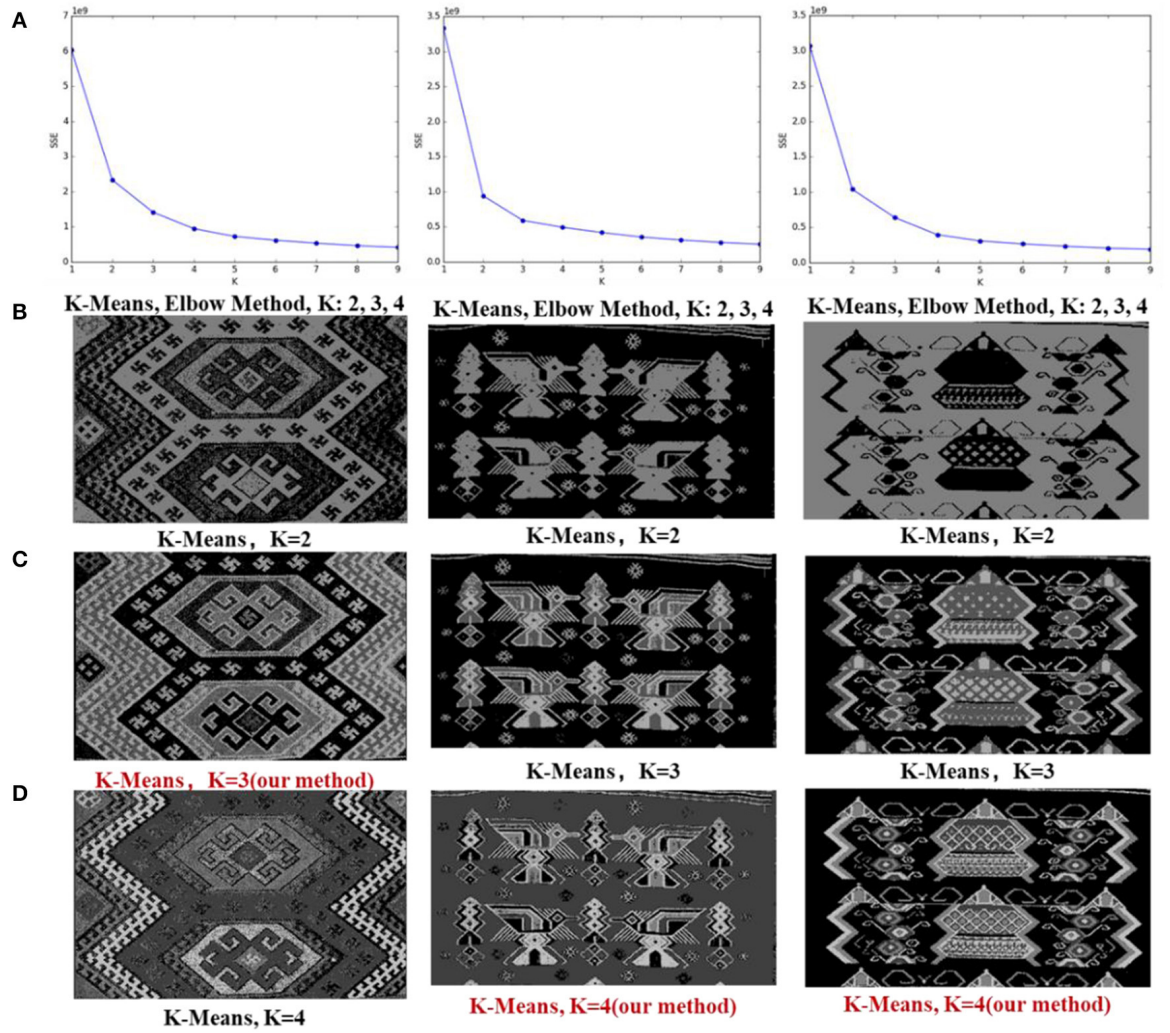
**(A)** Elbow method curve diagram; **(B–D)** *K*-means clustering results based on the *K*-values obtained by the elbow method. The red color represents the *K*-values calculated by the algorithm in this study.

In the experiment, the cluster value *K* is calculated by auto-selection. To verify whether the selection of the *K*-value produced by the algorithm is reasonable, the *K*-means algorithm is used to conduct an experimental comparison on 100 Tujia brocade pictures. Based on the index of the intra-cluster error variance [the sum of squared errors (SSE)] through the elbow method (Marutho et al., 2018), different *K*-values (*K* ∈ [2,9]) are selected to repeatedly train multiple *K*-means models to obtain relatively suitable clustering categories. The output values are then compared with the *K*-values calculated by the algorithm. Figure 8A displays the clustering SSE line graph obtained by the elbow method algorithm. As shown in Figure 8A, the optimal range of *k*-value is 2,3,4. Figures 8B–D shows the segmentation results of *k* = 2,3,4.

#### Calinski-Harabaz Index (CH)

For a clustering task, because the structure of the given dataset is unknown, the evaluation of the clustering results relies only on the characteristics and values of the dataset itself. Usually, the density within each cluster and the degrees of dispersion between clusters are used to evaluate the effect of clustering. Commonly used evaluation indicators are the silhouette coefficient (Luan et al., 2012) and CH (Liu et al., 2020). The CH is simple to calculate and runs much faster than the silhouette coefficient. Therefore, we choose the CH to evaluate the clustering effect of the approach. The CH calculation formula (13) (Liu et al., 2020) is as follows.

(13) $$\begin{array}{l} CH\left(k\right)=\frac{trB\left(k\right)/\left(k-1\right)}{trW\left(k\right)/\left(n-k\right)} \end{array}$$

where *n* represents the number of clusters, *k* represents the current class, *trB(k)* represents the trace of the inter-class dispersion matrix, and *trW(k)* represents the trace of the intra-class dispersion matrix. The larger the CH, the tighter the class itself, and the more scattered the classes, better clustering results are obtained.

In the experiment, the CH is calculated based on 156 Tujia brocades, and GMM is used to calculate the CH value of each cluster from *k* = *2* to *k* = *9*. The *K*-value rankings of our method are shown in Table 1.

**Table 1:** Calinski-Harabaz index (CH) ranking table.

**CH ranking**	**1**	**2**	**3**	**4**	**5**	**6**	**7**	**8**
Our method (sheet)	61	12	14	10	14	9	16	20

The commonly used clustering value selection methods and the method in this article are compared in terms of their running times and are shown in Table 2.

**Table 2:** The calculation time of the cluster value *K*.

**Algorithm**	**Elbow method**	**Calinski-Harabaz**	**Our method**
Time to calculate *K*-value (sheet)	40.17 s	56.05 s	0.22 s

Among them, the CH score for the *K*-value calculated by the method is the highest at 61. However, the highest CH score does not necessarily correspond to the best visual effect due to the particularity of the Tujia brocade dataset.

### Cluster Segmentation and Optimization Results

It was found through the experiments that *K*-means clustering is extremely sensitive to the choice of the *K*-value; *K*-means is also sensitive to noise points. The clustering effect is very good when the image background is clear and monotonous, but the clustering effect is not very good if the optimal cluster value *K* is not chosen or the image background texture is not obvious. Comparing the experimental results, it is found that GMM is more robust to the dataset than other models. As long as a suitable *K*-value range is chosen, the clustering effect is improved and the background texture characteristics have relatively little effect on the clustering results. From the perspective of the entire dataset, the GMM clustering effect is better than the *K*-means effect on the whole dataset.

**Figure 9:**
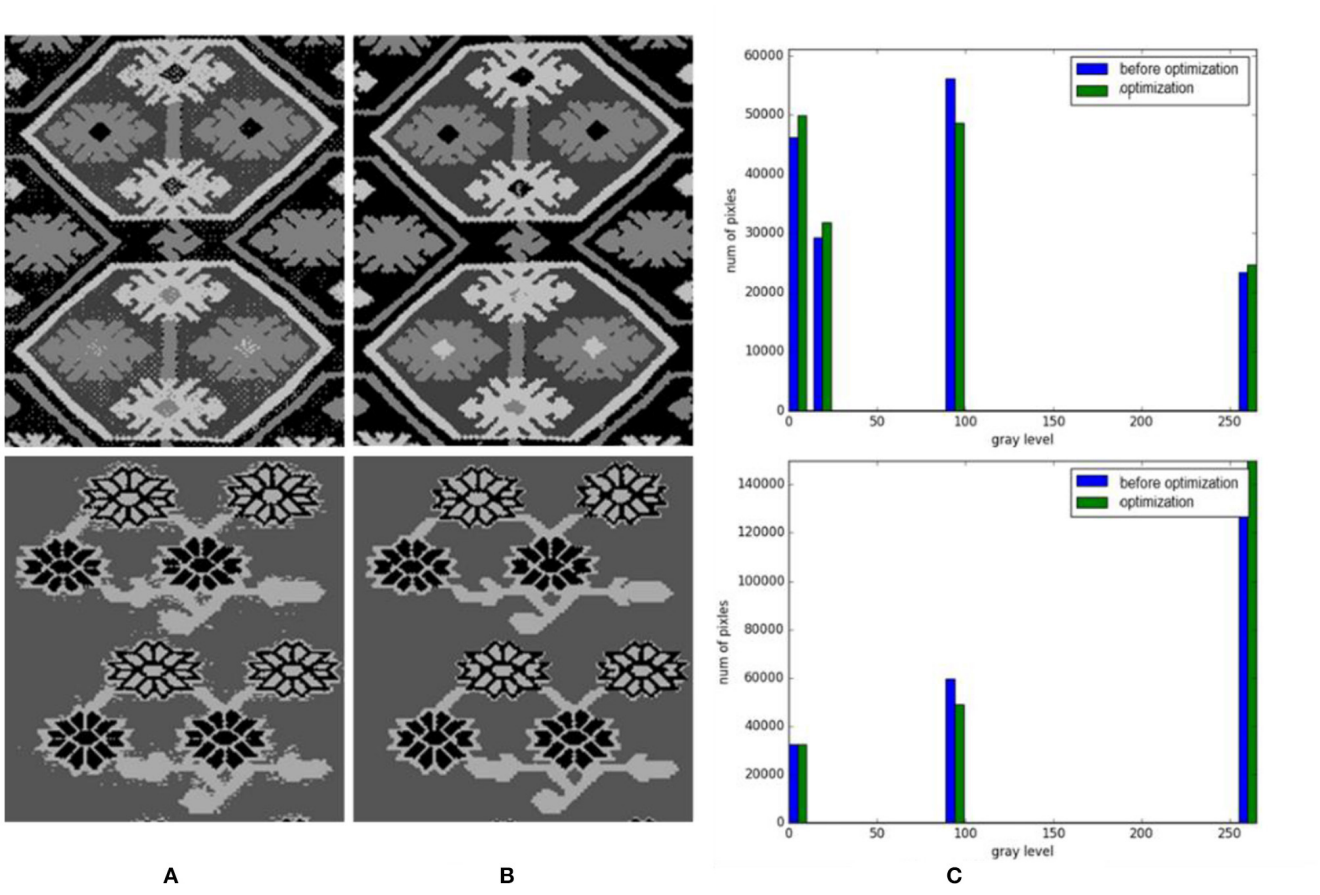
Gray histograms of cluster contrast optimization. **(A)** Before optimization. **(B)** Optimization. **(C)** Gray histograms.

Due to the particularity of Tujia brocade material and the brocade process, some noise points are formed after image clustering that affects the segmentation results. Therefore, we optimize the results after image clustering and compare the greyscale histograms before and after image optimization. The results are shown in Figure 9.

**Figure 10:**
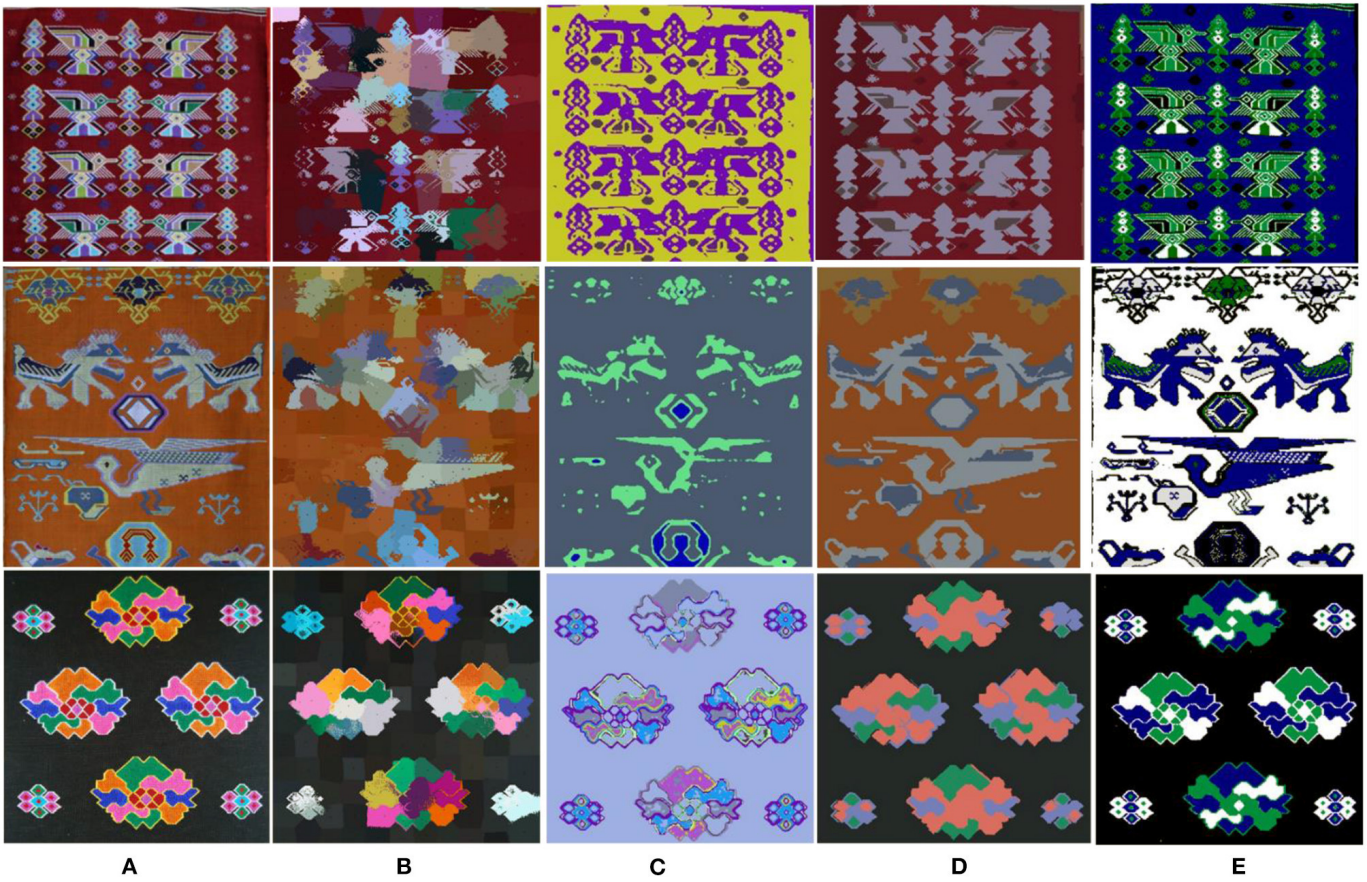
Contrast classic image segmentation algorithms and the method. **(A)** Original. **(B)** SLIC. **(C)** DCN. **(D)** Unsupervised Image Segmentation. **(E)** Our method.

We adopted some classic image segmentation algorithms, such as SLIC (Achanta et al., 2012), DCN (Yang et al., 2017), and Unsupervised Image Segmentation (Kanezaki, 2018), and the algorithm proposed in this study for the image segmentation of Tujia brocade. The segmentation results are shown in Figure 10. It was revealed that image information was lost by the segmentation based on a convolutional neural network as shown in Figures 10C,D.

**Figure 11:**
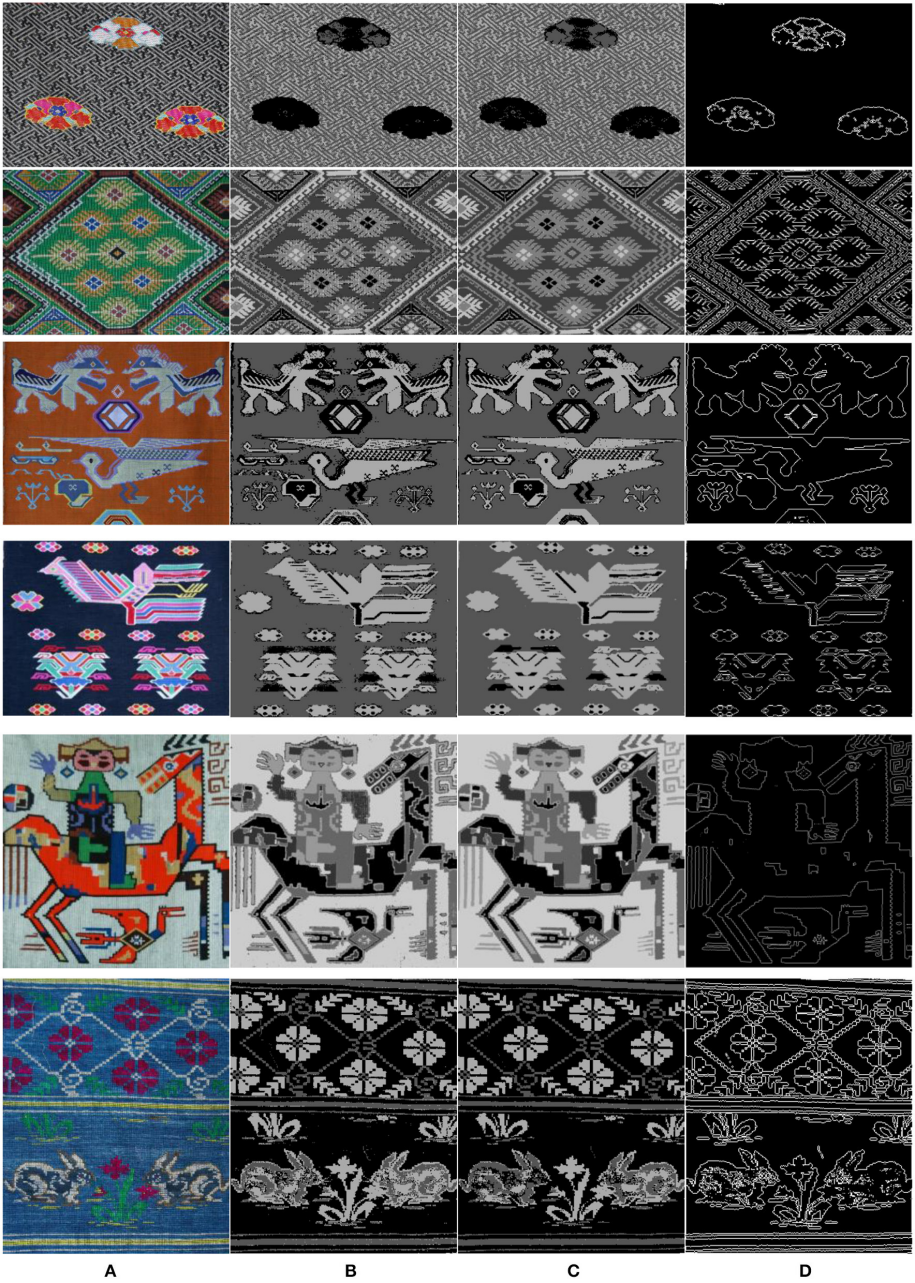
Clustering and optimization results of Tujia brocades. **(A)** Image: Original. **(B)** Cluster. **(C)** Optimization. **(D)** Mask.

Figure 11A is the original picture, Figures 11B,C show the clustering and optimization results of some Tujia brocades. After the clustering and optimization processes are completed, the required object mask is extracted, and the specific result is shown in Figure 11D.

**Figure 12:**
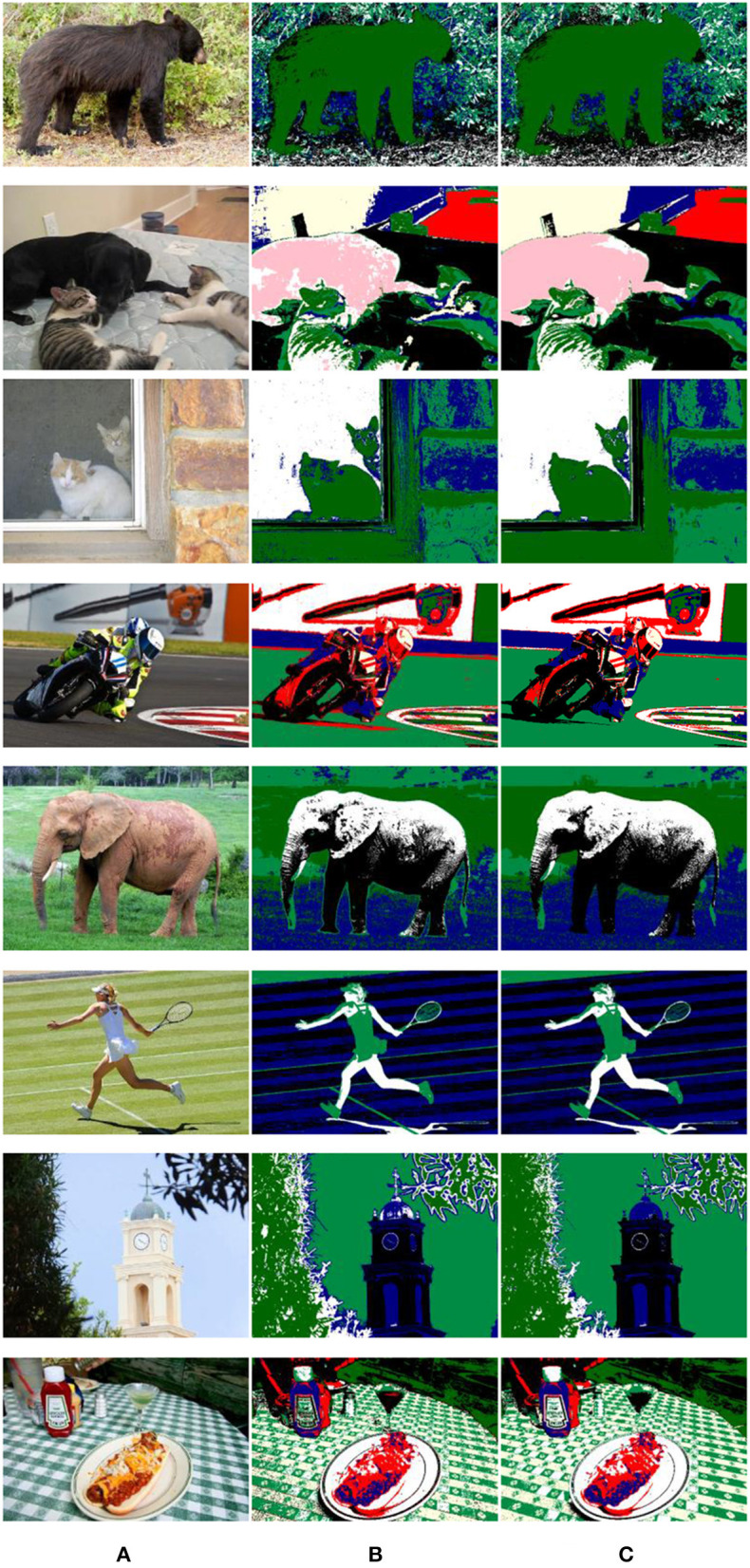
Clustering and optimization results of natural scene pictures. **(A)** Image: Original. **(B)** Cluster. **(C)** Optimization.

Figure 12A is a randomly selected picture from the Microsoft Common Objects in Context (MS COCO) dataset. The *K*-value of the cluster is calculated by the algorithm proposed in this article, and then the GMM is used for clustering. The result is shown in Figure 12B. The images in the MS COCO dataset are all high-definition pictures, and there is less interference from noise points, so only the dense conditional random field (DenseCRF) method is used in the optimization process, and the result is shown in Figure 12C.

## Conclusion

Due to the lack of a segmentation dataset for Tujia brocades, this article uses an unsupervised clustering method to segment Tujia brocades. Due to the rough textures of Tujia brocade patterns, the clustering results are more sensitive to the *K*-value, so we propose a *K*-value auto-selection algorithm based on a GLCM and LBPs. This method can quickly and effectively calculate a suitable *K*-value, and the speed is close to 100 times that of the elbow method and the CH approach. At the same time, an optimization method based on voting is proposed for the noise points generated after the clustering of the Tujia brocades. An experiment proved that the new method is remarkably effective for eliminating isolated noise points. Unsupervised clustering did not perform image segmentation semantically, so the clustered image needed post-processing to merge the clustered regions to form a whole segmentation object. Clustering-based image segmentation has high computational efficiency, but it is difficult to achieve image semantic segmentation because this method is based on low-level features of the image. In follow-up work, we plan to design an unsupervised image segmentation model by combining clustering with deep learning. It will use the feature extracted by a CNN for clustering, the clustering category labels as supervision information, and complete end-to-end Tujia brocade semantic segmentation.

## Data Availability Statement

The raw data supporting the conclusions of this article will be made available by the authors, without undue reservation.

## Author Contributions

The author confirms being the sole contributor of this work and has approved it for publication.

## Conflict of Interest

The author declares that the research was conducted in the absence of any commercial or financial relationships that could be construed as a potential conflict of interest.

## Publisher's Note

All claims expressed in this article are solely those of the authors and do not necessarily represent those of their affiliated organizations, or those of the publisher, the editors and the reviewers. Any product that may be evaluated in this article, or claim that may be made by its manufacturer, is not guaranteed or endorsed by the publisher.
